# A Vitamin E-Enriched Antioxidant Diet Interferes with the Acute Adaptation of the Liver to Physical Exercise in Mice

**DOI:** 10.3390/nu10050547

**Published:** 2018-04-28

**Authors:** Miriam Hoene, Martin Irmler, Johannes Beckers, Martin Hrabě de Angelis, Hans-Ulrich Häring, Cora Weigert

**Affiliations:** 1 Division of Pathobiochemistry and Clinical Chemistry, Department of Internal Medicine IV, University Hospital Tübingen, 72076 Tübingen, Germany; miriam.hoene@med.uni-tuebingen.de (M.H.); hans-ulrich.haering@med.uni-tuebingen.de (H.-U.H.); 2 Institute of Experimental Genetics, Helmholtz Zentrum München, German Research Center for Environmental Health (GmbH), 85764 Neuherberg, Germany; martin.irmler@helmholtz-muenchen.de (M.I.); beckers@helmholtz-muenchen.de (J.B.); hrabe@helmholtz-muenchen.de (M.H.d.A.); 3 Technische Universität München, Chair of Experimental Genetics, 85354 Freising, Germany; 4 German Center for Diabetes Research (DZD), 85764 Neuherberg, Germany; 5 Institute for Diabetes Research and Metabolic Diseases of the Helmholtz Zentrum München at the University of Tübingen, 72076 Tübingen, Germany

**Keywords:** liver, exercise, vitamin E, tocopherol, Ppargc1a, inflammation, lipogenesis

## Abstract

Physical exercise is beneficial for general health and is an effective treatment for metabolic disorders. Vitamin E is widely used as dietary supplement and is considered to improve non-alcoholic fatty liver disease by reducing inflammation and dyslipidemia. However, increased vitamin E intake may interfere with adaptation to exercise training. Here, we explored how vitamin E alters the acute exercise response of the liver, an organ that plays an essential metabolic role during physical activity. Mice fed a control or an α-tocopherol-enriched diet were subjected to a non-exhaustive treadmill run. We assessed the acute transcriptional response of the liver as well as glucocorticoid signalling and plasma free fatty acids (FFA) and performed indirect calorimetry. Vitamin E interfered with the exercise-induced increase in FFA and upregulation of hepatic metabolic regulators, and it shifted the transcriptional profile of exercised mice towards lipid and cholesterol synthesis while reducing inflammation. Energy utilization, as well as corticosterone levels and signalling were similar, arguing against acute differences in substrate oxidation or glucocorticoid action. Our results show that high-dose vitamin E alters the metabolic and inflammatory response of the liver to physical exercise. The interference with these processes may suggest a cautious use of vitamin E as dietary supplement.

## 1. Introduction

The beneficial effects of exercise make it a powerful means to improve general health and treat conditions such as obesity, non-alcoholic fatty liver disease (NAFLD), insulin resistance, and type 2 diabetes [1,2,3]. Physical exercise is a physiological, yet metabolically demanding, state that involves not only the contracting muscles, but the organism as a whole. The liver, an organ with pleiotropic functions, takes a particularly active role as it provides glucose to compensate for the increased uptake by the muscles [3,4]. This process is supported by a fall in hepatic energy charge as well as increased uptake and oxidation of fatty acids by the liver [3].

Increased mitochondrial substrate oxidation during exercise may result in the production of reactive oxygen species (ROS), a phenomenon that has long been known to occur in skeletal muscle [5]. Concern about the potential negative effects of oxidative stress has contributed to the propagation of antioxidant intake among athletes [5]. However, ROS, while possibly detrimental and even performance-limiting [6], may at the same time be essential signalling molecules which trigger antioxidant defenses and other beneficial long-term effects of exercise [7,8,9]. This hypothesis has been tested in different human and animal studies, frequently employing the vitamin E form α-tocopherol as an antioxidant and predominantly in combination with other antioxidative supplements. In human subjects, these studies have reported an interference with the training-induced improvement in insulin sensitivity [10] and blunted increases in transcriptional regulators of mitochondrial biogenesis and in mitochondrial proteins in skeletal muscle [10,11]. Similar effects of vitamin E have been described in rodents [12].

In addition to its local actions, some of which may be non-antioxidant activities [13], vitamin E could also interact with the endocrine response to physical exercise: It has been shown to reduce plasma corticosterone in stress-exposed rats [14] and to affect glucocorticoid signalling in skeletal muscle [15]. In humans, a combined vitamin E and C supplementation blunted the exercise-induced increase in cortisol [16]. While not all studies have confirmed the negative consequences of vitamin E-containing antioxidant supplements on training adaptation [17,18], a potential negative effect is certainly worth considering in view of the high prevalence of antioxidant supplementation: In a study published in 2005, more than 10% of US adults reported consuming at least 400 IU of vitamin E per day [19].

Similar to skeletal muscle, the liver shows both an acute stress response to physical exercise [20,21] and long-term adaptations to this metabolic challenge. We have previously reported that exercise acutely upregulates genes involved in the immune response and glucose and fatty acid metabolism [20], such as peroxisome proliferative activated receptor gamma coactivator 1 alpha (Ppargc1a), pyruvate dehydrogenase kinase 4 (Pdk4), insulin receptor substrate 2 (Irs2), angiopoietin like 4 (Angptl4) and the carnitine transporter solute carrier family 22 member 5 (Slc22a5), whereas it downregulates fatty acid synthase (Fasn) [22,23]. By increasing mitochondrial fatty acid oxidation and suppressing lipid anabolic processes in the liver, training reduces hepatic fat content and improves NAFLD, even in the absence of weight loss [3,23]. These direct effects on the liver may be a highly relevant mechanism by which physical activity improves general health. In contrast to the important roles of the liver in relation to physical exercise, little attention has been paid to whether its adaptation to exercise is also affected by vitamin E. This is particularly noteworthy as the liver is the main organ involved in the control of vitamin E status [24]. Vitamin E could affect the hepatic response to exercise by exerting local antioxidant or non-antioxidant activities [13], but also by interfering with glucocorticoid signalling [13,14,15], which has been implicated in the upregulation of Ppargc1a [25], Angptl4 [26], Irs2, and Pdk4 [27].

To study how vitamin E interacts with the acute hepatic adaptation to exercise, we fed mice an α-tocopherol-enriched antioxidant diet before subjecting them to a non-exhaustive treadmill run. We investigated the metabolic responses and changes in the liver transcriptome immediately after the bout of exercise. Vitamin E supplementation counteracted the increase in circulating free fatty acids (FFA) and the induction of inflammatory transcripts in the liver. At the same time, genes involved in cholesterol and lipid synthesis, processes that are normally suspended during physical exercise and negatively regulated by fatty acids, were upregulated by exercise combined with vitamin E. These effects appeared not to be related to alterations in glucocorticoid signalling or whole-body energy consumption in exercising mice. By altering the acute response to physical exercise, high doses of vitamin E could interfere with the beneficial long-term adaptation of the liver during regular exercise training.

## 2. Materials and Methods

### 2.1. Diets and Exercise Protocol

The animal experiment was performed in accordance with the EU Directive 2010/63/EU and approved by the local authorities (Regierungspräsidium Tübingen). Mice were kept under an inverted light–dark cycle (dark period 9:30–21:30 h, light period 21:30–9:30 h). Experiments were performed between 10:00 and 14:00 h. Seven week-old male C57BL/6N mice were purchased from Charles River (Sulzfeld, Germany) and assigned to either a diet supplemented with 100 mg/kg vitamin C and 2000 IU/kg vitamin E (as α-tocopheryl acetate) or to control diet (0 mg/kg vitamin C, 149 IU/kg vitamin E) based on the purified diet C1000 (Altromin, Lage, Germany). In contrast to humans, mice can synthetize vitamin C. However, a small amount of vitamin C was added to the vitamin E-enriched diet since it is required for vitamin E regeneration [28].

After 4 weeks of feeding, at an age of 11–12 weeks, the animals were weighed and *n* = 12 mice from each dietary group were subjected to 1 h of treadmill running (13 m/min and 14° uphill slope), as described previously [22]. For a subset (*n* = 6 per diet) of exercising mice, respirometry was performed during the treadmill run using an indirect calorimetric system (Oxylet, Panlab, Cornellà, Spain). Oxygen consumption and the respiratory quotient (CO_2produced_/O_2consumed_) were calculated using Metabolism 2.1.04 Software (Panlab, Cornellà, Spain). Immediately after the exercise bout, mice were analgosedated with an intraperitoneal injection of ketamine and xylazine (150 and 10 mg/kg body weight, respectively), and exsanguinated by decapitation. Blood was transferred into EDTA collectors and livers were removed and frozen in liquid nitrogen. Blood and liver collection was carried out within 5 min of completion of the exercise bout. The control mice (*n* = 10 per diet) remained in their cages, but in close proximity to the treadmill, and were deprived of food for 60 min before they were killed, to align with the feeding state of the exercised animals.

### 2.2. Metabolites and Glucocorticoid Receptor Activity

Plasma FFA concentrations were determined using an enzyme-based colorimetric kit (HR Series NEFA-HR(2), Wako Chemicals GmbH, Neuss, Germany) on an automated clinical chemistry analyzer (ADVIA 1650, Siemens Healthcare Diagnostics, Fernwald, Germany). Vitamin E was analyzed on a HPLC with UV detection; sample preparation and analysis were performed using a commercial kit (Vitamins A and E in Serum/Plasma, 34000, Chromsystems, München, Germany) according to the manufacturer’s instructions. Plasma corticosterone was measured by radioimmune assay (Double Antibody Corticosterone ^125^I RIA Kit for Rats and Mice, 07120103, MP Biomedicals, Santa Ana, CA, USA; coefficients of variation according to manufacturer: intra-assay 4–10% for 56–370 ng/mL, inter-assay 6–7% for 119–469 ng/mL).

Quantification of triglycerides was done by homogenizing frozen liver pieces in 0.9% NaCl containing 1% Triton X-100 (10 µL/mg tissue) using a TissueLyser (Qiagen, Hilden, Germany). Debris was removed by centrifugation (10 min, 16,000 g, room temperature), and triglycerides in the supernatant were quantified on an automated clinical chemistry analyzer using an enzyme-based colorimetric kit (ADVIA Chemistry TRIG_2, Siemens Healthcare Diagnostics). Glucocorticoid receptor DNA binding activity was assessed with the TransAM GR DNA-binding ELISA (45496, Active Motif, Carlsbad, CA, USA) using nuclear extracts prepared from frozen liver tissue using the NE-PER™ Nuclear and Cytoplasmic Extraction Kit (78833, ThermoFisher, Waltham, MA, USA), in accordance with the manufacturers’ instructions.

### 2.3. RNA Isolation, Quantitative PCR and Transcriptome Analysis

RNA isolation, reverse transcription and quantitative real-time PCR were performed as previously described [22] using QuantiTect Primer Assays (Qiagen, Hilden, Germany). The mRNA content is given in arbitrary units. The non-normalized results shown throughout the manuscript were similar after normalization to β-actin mRNA (not shown).

Transcriptome analysis was done as in [29], with the following differences: we used Mouse Gene 2.1 ST arrays (Affymetrix, Santa Clara, CA, USA) and Expression Console software (v.1.4.1.46, Affymetrix). Array data have been submitted to the GEO database at NCBI (GSE110747).

### 2.4. Data Analysis

Statistical analysis was performed with JMP 13 (SAS Institute, Cary, NC, USA). Plasma analytes, glucocorticoid activity and weight were compared by Student’s *t*-tests. mRNA data were compared using the non-parametric Wilcoxon method as they did not fulfill the normality requirement. A *p*-value < 0.05 was considered significant and *p* < 0.1 was considered a trend. Data are shown as means ± standard deviation (SD). 

Microarray data were analyzed as described [29] with limma *t*-tests and a *p*-value < 0.05 as the cut-off. To reduce the background signal, data were filtered for average linear arbitrary expression >4 in at least one group. Transcripts with a limma *t*-test *p*-value < 0.05 and median fold change >|1.2| between exercised mice fed the control or the vitamin E diet were subjected to upstream regulator analysis using Ingenuity Pathway Analysis (Qiagen, Redwood City, CA, USA). The upstream regulator types drugs and chemicals were excluded. Open-source MultiExperiment Viewer software [30] was employed for heatmap generation, using autoscaled values. 

## 3. Results

### 3.1. Vitamin E Interferes with Exercise-Induced Increase in Plasma FFA and Expression of Key Metabolic Regulators in the Liver

**Figure 1:**
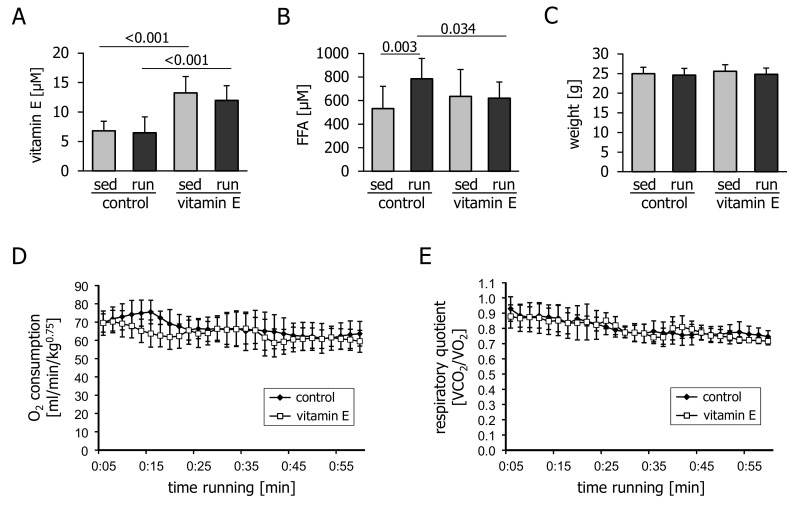
Plasma levels of (**A**) vitamin E and (**B**) circulating free fatty acids (FFA) in mice immediately after 1 h of treadmill running (run) or remaining sedentary (sed) and fasting for the same period of time. Prior to the experiment, mice had been fed a control or a vitamin E-enriched diet for four weeks. (**C**) Body weight, measured before the exercise bout; (**D**) O_2_ consumption and (**E**) respiratory quotient measured during the treadmill run. Values are means ± standard deviation from *n* = 10 (sedentary groups) or *n* = 12 (exercised groups) animals (**A**–**C**) or from *n* = 6 mice (**D**,**E**).

Dietary supplementation of α-tocopherol resulted in an approximately two-fold increase in the concentration of plasma vitamin E that was not changed by the acute treadmill run (Figure 1A). Exercise caused a moderate increase in plasma FFA levels that was blunted by vitamin E supplementation (Figure 1B). Body weight was similar in all groups (Figure 1C). The O_2_ consumption (Figure 1D) and respiratory quotient (Figure 1E) of running mice were not affected by vitamin E intake, indicating a similar reliance on fatty acids as a metabolic fuel.

**Figure 2:**
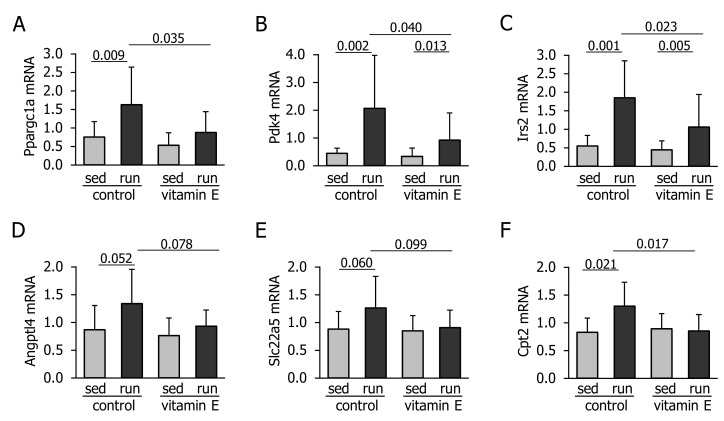
Liver mRNA levels of (**A**) peroxisome proliferative activated receptor gamma coactivator 1 alpha (Ppargc1a), (**B**) pyruvate dehydrogenase kinase 4 (Pdk4), (**C**) insulin receptor substrate 2 (Irs2), (**D**) angiopoietin like 4 (Angptl4), (**E**) solute carrier family 22 member 5 (Slc22a5), and (**F**) carnitine palmitoyltransferase 2 (Cpt2) in mice immediately after 1 h of treadmill running (run) or remaining sedentary (sed) and fasting for the same period of time. Prior to the experiment, mice had been fed a control or a vitamin E-enriched diet for four weeks. Values are means ± standard deviation from *n* = 10 (sedentary groups) or *n* = 12 (exercised groups) animals in arbitrary units.

Exercise caused the upregulation of Ppargc1a Pdk4, Irs2, Angptl4, Slc22a5 and carnitine palmitoyltransferase 2 (Cpt2) (Figure 2A–F). Vitamin E significantly reduced the upregulation of Ppargc1a, Pdk4, Irs2 and Cpt2 and tended to lower the mRNA levels of Angptl4 and Slc22a5 (Figure 2A–F).

### 3.2. Corticosterone Levels and Glucocorticoid Signalling are not Affected by Vitamin E

**Figure 3:**
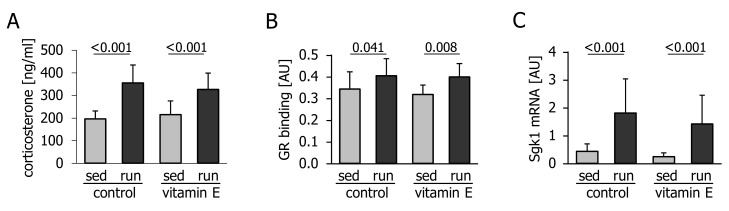
(**A**) Plasma corticosterone levels, (**B**) glucocorticoid receptor (GR) DNA-binding activity, and (**C**) amount of liver serum/glucocorticoid regulated kinase 1 (Sgk1) mRNA in mice immediately after 1 h of treadmill running (run) or remaining sedentary (sed) and fasting for the same period of time. Prior to the experiment, mice had been fed a control or a vitamin E-enriched diet for four weeks. Values are means ± standard deviation from *n* = 10 (sedentary groups) or *n* = 12 (exercise groups) animals. AU, arbitrary units.

The attenuated upregulation of the exercise-responsive transcripts Ppargc1a, Pdk4, Irs2, and Angptl4 might have been due to the previously reported interference of vitamin E with glucocorticoid levels and signalling. In our study, exercise caused a pronounced increase in circulating corticosterone levels (Figure 3A), a significant increase in glucocorticoid receptor DNA-binding activity (Figure 3B) and upregulation of the glucocorticoid receptor target gene serum/glucocorticoid regulated kinase 1 (Sgk1), in the liver (Figure 3C). However, there was no effect of vitamin E (Figure 3A–C).

### 3.3. Vitamin E Alters the Hepatic Transcriptional Response Related to Lipid and Cholesterol Synthesis and Inflammation

To gain a more detailed insight into how vitamin E alters the regulatory processes that normally take place in the liver in response to exercise, we next performed a global transcriptome analysis. A total of 445 transcripts were significantly different (*p* < 0.05, median fold change >|1.2|) between exercised vitamin E-supplemented and exercised control mice. Among the 143 upregulated transcripts, only one, the phospholipid transfer protein (Pltp), was also upregulated by vitamin E in the sedentary state (median fold increase 1.58 in sedentary and 1.43 in exercised mice). Among the 302 downregulated transcripts, only two protein-coding genes showed consistent downregulation due to vitamin E supplementation in both sedentary and exercised mice: RNA specific adenosine deaminase (Adar) and the interleukin 21 receptor (Il21r). Next, all 445 transcripts differentially expressed between exercised vitamin E-supplemented and exercised control mice were subjected to an upstream regulator analysis using Ingenuity Pathway Analysis. A total of 11 upstream regulators were predicted to be activated (*z*-score ≥ 2) and 20 to be inhibited (*z*-score ≤ −2) with a significant *p*-value of overlap (*p* < 0.05) (Table 1).

**Figure 4:**
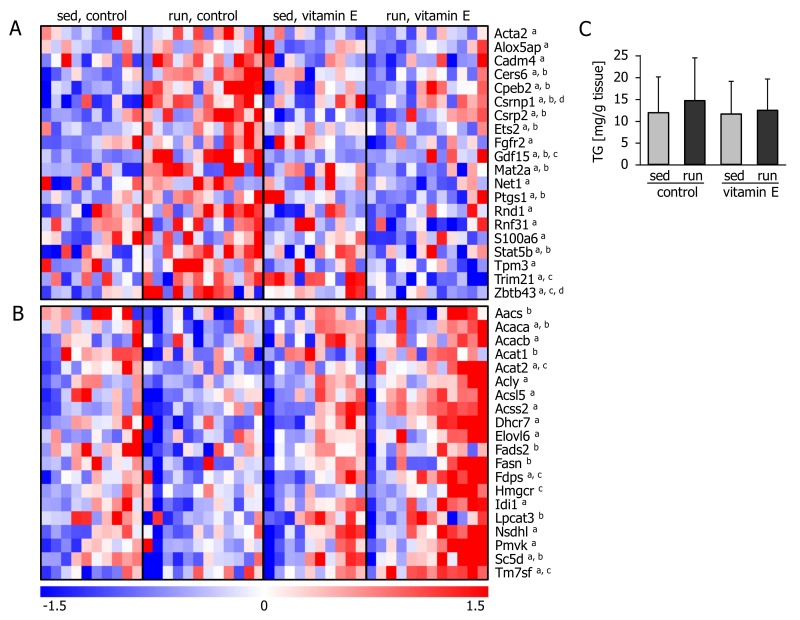
Liver transcripts associated with upstream regulators of (**A**) inflammation and (**B**) lipid and cholesterol synthesis. Each column represents one animal. Superscript letters denote significant differences (*p* < 0.05) between ^a^ exercised mice fed the control or the vitamin E-enriched diet, ^b^ sedentary and exercised control mice, ^c^ sedentary and exercised vitamin E-supplemented mice, and ^d^ sedentary mice fed a control or vitamin E-enriched diet. (**C**) Liver triglycerides (TG) of mice immediately after 1 h of treadmill running (run) or remaining sedentary (sed) and fasting for the same period of time. Prior to the experiment, mice had been fed a control or a vitamin E-enriched diet for four weeks. Values are means ± standard deviation from *n* = 10 (sedentary groups) or *n* = 12 (exercise groups) animals.

The upstream regulators predicted to be inhibited by vitamin E after the exercise bout comprised a number of well-known inflammatory players: toll like receptor 7 (TLR7) and the TLR adaptor protein, TICAM1; alpha, beta, and gamma interferons; transforming growth factor beta 1 (TGFB1) and the cytokines colony stimulating factor 2 (CSF2) and oncostatin (OSM), as well as the transcription factor cAMP responsive element binding protein 1 (CREB1) which may also be involved in immune function [31]. Next, we took a closer look at the individual transcripts predicted to be regulated by these inflammatory players according to Ingenuity Pathway Analysis. Several of these transcripts, such as growth differentiation factor 15 (Gdf15), prostaglandin synthase 1 (Ptgs1), and signal transducer and activator of transcription 5B (Stat5b), were increased in the livers of exercising control-fed mice, and this inflammatory response was attenuated by vitamin E supplementation (Figure 4A).

Among the 11 transcriptional regulators predicted to be activated in the vitamin E group, we identified six as being involved in lipid synthesis and cholesterol metabolism: the nuclear receptor subfamily 1 receptors (Nr1h, also known as LXR), peroxisome proliferator activated receptor delta (PPARD), MLX interacting protein like (MLXIPL, also known as ChREBP), the sterol regulatory element binding transcription factors (SREBF) 1 and 2, and SCAP, a protein with a sterol sensing domain that activates SREBFs in the absence of cholesterol. Complementing these activated factors, INSIG, which acts antagonistically to SREBF1/2 and SCAP, was predicted to be inhibited (Table 1).

The transcript levels of key enzymes involved in lipogenesis and cholesterol synthesis, such as Fasn, acetyl-CoA carboxylases alpha (Acaca) and beta (Acacb),ATP citrate lyase (Acly), 3-hydroxy-3-methylglutaryl-CoA reductase (Hmgcr) and farnesyl diphosphate synthase (Fdps), were expressed at similar or even lower levels in the livers of sedentary and of exercised control-fed mice (Figure 4B). In contrast, in the livers of exercised, vitamin E-supplemented mice, the amount of these transcripts was generally increased. None of these transcripts showed a significant difference between the sedentary groups. This apparent exercise-induced activation of lipogenic pathways in vitamin E-supplemented mice was not reflected by a different hepatic triglyceride content at the investigated timepoint—immediately after the exercise bout (Figure 4C).

## 4. Discussion

The results of our study show that vitamin E (α-tocopherol) supplementation can impair the acute adaptation of the liver to physical exercise. The hepatic exercise response is characterized by an increase in glucose production and fatty acid oxidation [3] and by an activation of inflammatory and stress signalling pathways [19,20]. We have previously reported that the liver responds to exercise with an increased transcription of key regulators of mitochondrial biogenesis and glucose and lipid metabolism [21]. In the long run, these acute responses are supposed to translate into decreased lipogenesis and increased lipid oxidation, which both contribute to the reduction of hepatic fat content [3]. The results of our global transcriptome analysis and targeted mRNA quantification indicate that vitamin E supplementation interferes with the acute metabolic and inflammatory responses of the liver. At the same time, vitamin E counteracted the moderate increase in circulating FFA that is normally seen shortly after an endurance activity of this type in mice [21] and humans [32].

An Ingenuity Pathway Analysis comparing exercised mice with and without vitamin E supplementation predicted the activation of 11 upstream regulators, while 20 were inhibited. Out of the 11 activated factors, six were transcriptional regulators of lipid and cholesterol synthesis, such as SREBP, SCAP, and ChREBP. In contrast, INSIG, a negative regulator of SREBP and SCAP, was predicted to be inhibited. When looking at individual transcripts that are under the control of these regulators, we found the rate-limiting enzymes in fatty acid synthesis to be significantly higher in exercised, vitamin E-fed mice than in exercised control mice, for example, the levels of Fdps and of Acaca and Acacb. Acacb and the fatty acid synthase, Fasn, were downregulated by exercise under control diet conditions, and this downregulation was abrogated with vitamin E. In contrast, vitamin E supplementation caused transcripts such as Fdps and Hmgcr, which are key enzymes in cholesterol biosynthesis, to be upregulated by exercise. Thus, vitamin E supplementation shifted the overall lipid and cholesterol synthesis-related transcriptional response towards an activation that is normally not seen after physical activity.

Since the upstream regulators SREBP, LXR and ChREBP are normally inhibited in catabolic conditions by fatty acids [33], the different transcriptional profiles could, at least in part, be related to the attenuated exercise-induced increase in FFA levels in vitamin E-fed mice. These lower FFA concentrations could be caused by a reduced rate of lipolysis or by an increased uptake and utilization. We did not detect differences in whole-body oxygen consumption, a measure of energy expenditure, or in the respiratory quotient, which reflects the percentage of fat oxidized. Therefore, the lower plasma FFA concentrations were likely not caused by major differences in the utilization of energy sources. FFA taken up by the liver could also be re-esterified into triglycerides. While we did not find liver triglyceride levels to be different, the increased abundance of Pltp mRNA in sedentary and exercised vitamin-E fed mice may be of interest in this context; Pltp is a vitamin E transfer protein and is also important for the lipidation and hepatic secretion of VLDL [34]. Thus, the vitamin E-supplemented mice may not have accumulated hepatic triglycerides due to increased VLDL release.

Alternatively, vitamin E could have reduced adipose tissue lipolysis through its previously reported interference with the glucocorticoid response [13,14,15], as glucocorticoids stimulate lipolysis [35]. Glucocorticoid receptor signal transduction has also been implicated in the gene expression of Irs2 and Pdk4 [27], Angptl4 [26], and Ppargc1a [25]. However, we could not find evidence for an altered glucocorticoid response as corticosterone levels and signalling increased to a similar extent in both groups of exercised mice. Catecholamine-triggered cAMP-dependent pathways are important activators of lipolysis as well as regulators of Irs2 [36] and Ppargc1a [37] transcription. A potential suppressive effect of vitamin E on cAMP signalling was suggested by the predicted inhibition of the upstream regulator CREB. We did not, however, attempt to quantify catecholamines, which increase rapidly in mice during procedures such as immobilization and anaesthesia.

The list of inhibited regulators further encompassed several inflammatory players, namely, TLR7, TGFB1, and the cytokines IFNB1, IFNG, CSF2 and OSM. Transcripts that were upregulated by exercise only in the absence of vitamin E included the TGFB family member and pleiotropic cytokine Gdf15, the cytokine signal transducer Stat5b, and enzymes involved in the synthesis of inflammation-related molecules, such as prostaglandin synthase (Ptgs1), arachidonate 5-lipoxygenase activating protein (Alox5ap) and ceramide synthase 6 (Cers6).

The detailed mechanism by which vitamin E altered the inflammatory response to exercise is unclear. Vitamin E is known to decrease pro-inflammatory cytokines such as interleukin 6 (IL-6) [38], which is known to acutely increase during physical exercise [39]. We did not assess IL-6 levels in this study, but we have previously found mice lacking IL-6 to respond to physical activity with similar increases in circulating FFA and in hepatic Ppargc1a and Irs2 transcription [40], which argues against a major role for circulating IL-6 in mediating the vitamin E effects of exercise. However, cytokines other than IL-6 may also play a role and be confined to a local response, and as vitamin E also interferes with monocyte adhesion, it could affect the inflammatory response independent of differences in cytokine levels [38].

An interesting question is whether the acute effects observed in this study translate to altered or impaired adaptation of the liver when physical exercise training is accompanied by high vitamin E intake, in particular as a means to improve insulin sensitivity and NAFLD. We are only aware of one study that has investigated the effects of training combined with a vitamin E-only supplementation on the liver. This study, performed in rats, found that vitamin E counteracted swim training-induced increases in Ppargc1a protein and mitochondrial content [41], indicating that at least some of the acute effects reported here can culminate in long-term impairments of the hepatic adaptation to exercise.

Vitamin E appeared to affect glucose and lipid metabolism-related transcription in a concerted fashion. Firstly, it counteracted the induction of factors involved in mitochondrial biogenesis (Ppargc1a), insulin signalling (Irs2), fatty acid oxidation (Cpt2), and metabolite fuel selection (Pdk4). Secondly, transcripts involved in lipid and cholesterol synthesis were increased in the condition involving exercise plus vitamin E, which is in contrast to the reduction in lipogenic enzymes in the liver observed in the exercised control mice and in previously published studies [22,42]. Thus, vitamin E could interfere with both mechanisms that mediate the reduction of liver fat following regular physical activity, with the downregulation of lipogenic enzymes and with the upregulation of enzymes involved in fatty acid oxidation.

According to the hormesis theory [7], acute, tolerable stress can be viewed as adjuvant stimulus that triggers beneficial long-term effects. The predominant part of exercise-related research published so far has dealt with skeletal muscle. Here, it is already known that besides redox signalling, a proper local inflammatory response is required for functional adaptation to exercise [43]. In this context, it is worth looking at how physical exercise modulates immune function on the systemic level: Acutely, exercise elicits an intensity-dependent cytokine response [39]. Regular training, in contrast, reduces systemic low-grade inflammation [44,45] and hepatic inflammation associated with NAFLD and obesity [46] and alters the immune state of the liver, making it less susceptible to acute TNF-mediated injury [47]. If, in analogy to the muscle, the acute inflammatory response of the liver contributes to a beneficial adaptation process, one might speculate that the interference of vitamin E with this response is not desirable.

Studies testing vitamin E supplementation for the treatment of NAFLD have not reached a clear conclusion regarding its efficacy and safety, and there is even debate over whether it may increase all-cause mortality at high doses [48]. One reason for the equivocal results of in vivo vitamin E studies might be the fact that it has different modes of action that are still not completely understood [49]. Our data suggest that some effects of vitamin E might only come into play during physical exercise or similar metabolic challenges.

## 5. Conclusions

Vitamin E affected the acute response of the liver to physical exercise in a concerted fashion that was potentially related to lower levels of circulating FFA. The differences in FFA concentrations and in hepatic transcription were not caused by the previously-reported inhibition of glucocorticoid signalling by vitamin E. By dampening the inflammatory and altering the metabolic response, increased vitamin E intake could interfere with the beneficial adaptations that normally occur in the liver as a result of physical exercise training.

## Figures and Tables

**Table 1:** Ingenuity upstream regulator analysis of the 445 transcripts that were significantly different between exercised mice fed a control or a vitamin E-enriched diet (limma *t*-test *p* < 0.05 and median fold change >|1.2|). All regulators with *z*-score ≥|2.0| (i.e., predicted to be activated or inhibited) and with a *p*-value of overlap <0.05 are shown (drugs and chemical upstream regulators were excluded).

Upstream Regulator	Molecule Type	Activation *z*-Score	*p*-Value of Overlap	Predicted State
SCAP	Other	3.58	<0.001	Activated
SREBF2	Transcription regulator	3.27	<0.001	Activated
SREBF1	Transcription regulator	2.71	<0.001	Activated
TSC2	Other	2.45	0.004	Activated
Nr1h (LXR)	Group	2.40	0.016	Activated
PPARD	Ligand-dependent nuclear receptor	2.23	0.006	Activated
MLXIPL (ChREBP)	Transcription regulator	2.22	<0.001	Activated
miR-145-5p ^1^	Mature microRNA	2.20	<0.001	Activated
FAS	Transmembrane receptor	2.18	0.004	Activated
INHA	Growth factor	2.00	0.049	Activated
ATP7B	Transporter	2.00	0.002	Activated
TLR7	Transmembrane receptor	−2.62	0.004	Inhibited
Interferon alpha	Group	−2.61	0.031	Inhibited
TGFB1	Growth factor	−2.59	0.002	Inhibited
TICAM1	Other	−2.43	0.011	Inhibited
SP1	Transcription regulator	−2.43	0.033	Inhibited
APP	Other	−2.40	0.024	Inhibited
IFNB1	Cytokine	−2.40	0.013	Inhibited
INSIG1	Other	−2.39	<0.001	Inhibited
SRF	Transcription regulator	−2.37	0.002	Inhibited
Vegf	Group	−2.36	0.035	Inhibited
POR	Enzyme	−2.35	<0.001	Inhibited
MAP4K4	Kinase	−2.24	0.005	Inhibited
Ifn	Group	−2.20	0.021	Inhibited
CREB1	Transcription regulator	−2.17	0.008	Inhibited
TGM2	Enzyme	−2.16	<0.001	Inhibited
IFNG	Cytokine	−2.15	<0.001	Inhibited
MGEA5	Enzyme	−2.11	0.025	Inhibited
CSF2 (GM-CSF)	Cytokine	−2.07	0.018	Inhibited
OSM	Cytokine	−2.04	0.025	Inhibited
SAMSN1	Other	−2.00	0.032	Inhibited

Common alternative symbols are given in brackets. ^1^ And other miRNAs with the seed UCCAGUU.
